# Electrochemical Analysis for Enhancing Interface Layer of Spinel LiNi_0.5_Mn_1.5_O_4_ Using p-Toluenesulfonyl Isocyanate as Electrolyte Additive

**DOI:** 10.3389/fchem.2019.00591

**Published:** 2019-08-27

**Authors:** Zhe Xiao, Renheng Wang, Yan Li, Yiling Sun, Shuting Fan, Keyu Xiong, Han Zhang, Zhengfang Qian

**Affiliations:** College of Physics and Optoelectronic Engineering, Shenzhen University, Shenzhen, China

**Keywords:** lithium ion battery, LiNi_0.5_Mn_1.5_O_4_, p-toluenesulfonyl isocyanate, solid electrolyte interface, electrolyte additive

## Abstract

LiNi_0._5Mn_1.5_O_4_ (LNMO) is a potential cathode material for lithium-ion batteries with outstanding energy density and high voltage plateau (>4.7 V). However, the interfacial side reaction between LNMO and the liquid electrolyte seriously causes capacity fading during cycling at the high voltage. Here, p-toluenesulfonyl isocyanate (PTSI) is used as the electrolyte additive to overcome the above problem of LNMO. The results show that the specific capacity of LNMO/Li cell with 0.5 wt.% PTSI at the first cycle is effectively enhanced by 36.0 mAh/g and has better cycling performance than that without PTSI at 4.98 V. Also, a stable solid electrolyte interface (SEI) film derived from PTSI is generated on the electrode surface, which could alleviate the strike of hydrofluoric acid (HF) caused by electrolyte decomposition. These results are explained by the molecular structure of PTSI, which contains SO_3_. The S=O groups can delocalize the nitrogen nucleus to block the reactivity of PF_5_.

## Introduction

Over the past few years, the high energy and power density capability of lithium-ion batteries (LIBs) have been interested extremely, due to potential applications in electric vehicles (EVs), hybrid electric vehicles (HEVs), and plug-in hybrid electric vehicles (PHEVs) (Taracson and Armand, 2001; Armand and Tarascon, 2008; Ji et al., 2011; Kim et al., 2012). In order to improve the energy density and power density of batteries, a large number of Li compounds (e.g., olivine-type materials, silicates, Mn-rich, and Ni-rich layered materials) have been studied by researchers all over the world (Chen et al., 2014; Zhang et al., 2014; He et al., 2015a,b; Panchal et al., 2017; Chan et al., 2018; Li et al., 2018; Qiu et al., 2018). Spinel LiNi_0.5_Mn_1.5_O_4_ (LNMO) is a promising material to replace layered LiCoO_2_ as a cathode for high power density LIBs (Carlier et al., 2003; Su et al., 2017; Sun et al., 2018). LNMO has an high charge-discharge platform (>4.7 V) and outstanding cycling stability (Wang F. et al., 2017). Unfortunately, the high charging voltage (~4.7 V) is higher than the stable voltage of LiPF_6_-based electrolyte, resulting in rapid oxidation decomposition of the electrolyte and unnecessary secondary reactions at the LNMO/electrolyte interface (Li et al., 2013; Deng et al., 2017; Ma et al., 2019). Furthermore, hydrofluoric acid (HF) derives from hydrolysis of LiPF_6_-based electrolyte, which can dissolve Mn^3+^ from LNMO (Xiao et al., 2017). The Mn^3+^ dissolution into the electrolyte causes a cracking solid electrolyte interface (SEI) and reduces rapidly specific capacity, so the LNMO cell exhibits poor cycling peculiarity (Liu et al., 2017; Mou et al., 2018).

One way is to make electrolyte additives form a stable SEI film on the cathode, which inhibits LNMO electrode interface erosion and electrolyte decomposition, scavenging type to capture HF (Haregewoin et al., 2016; Wang et al., in press). As the strong acid produced from LiPF_6_ is considered the initiator which induces the cleavage and polymerization of cyclic carbonate under high voltage conditions, many researchers are trying to add some oxidation-resistant solvents, for example, sulfones (Hilbig et al., 2017; Su et al., 2017), nitriles (Abu-Lebdeh and Davidson, 2009) and fluoro solvents (Kim et al., 2017). However, when adding the oxidation-resistant solvents there are new problems, including a decrease of conductivity, an increase of viscosity and poor compatibility. Hence, a number of suitable functional additives, such as (pentafluorophenyl) diphenylphosphine (PFPDPP) (Bolloju et al., 2019), dimethyl phenylphosphonite (DMPP) (Mai et al., 2015), tris (trimethylsilyl) phosphite (TMSP) (Wang et al., 2016), triethyl borate (TEB) (Chen et al., 2017), lithium bisoxalatodifluorophosphate (LiBODFP) (Yang et al., 2019), and so on, have been developed to perform better of LIBs under high voltage. As previously reported (Wang R. H. et al., 2015), p-toluenesulfonyl isocyanate (PTSI) has excellent physical and chemical properties because of SO_3_ and S=O groups. What's more, the lowest unoccupied molecular orbital (LUMO) (−0.2469 Ha) occupied by PTSI is lower than that by vinylene carbonate (VC, LUMO = −0.2274 Ha) (Xu, 2004; Wu et al., 2012).

In this work, PTSI will be used as an additive for LiPF_6_-based electrolyte. The main direction of the experiment is to study the SEI film generated between electrolyte and LNMO electrode surface. We hope the PTSI can form a stable SEI film, suppress the corrosion of LNMO electrode by HF, reduce the formation of other products, and improve the circulation ability of LNMO battery at high voltage.

## Experimental

### Preparation of the Electrolyte

The basic electrolyte (Jiangxi Youli New Materials Co., Ltd., China) was a 1 M (M = mol/L) ethylene carbonate (EC)/ethyl carbonate (EMC)/diethyl carbonate (DEC) LiPF_6_-based electrolyte in 1:1:1 configuration. The desired concentration 0.5 wt.% of PTSI additive was achieved by dissolving the corresponding amount of PTSI in the base electrolyte and stirring for 5 min in an argon-filled glovebox, and the oxygen and water content were <1 ppm. The supernatant was measured using a Karl Fischer 831 Coulometer (Metrohm) for H_2_O and Karl Fischer 798 GPT Titrino (Metrohm) for HF, respectively.

### Electrochemical Characterization

LNMO electrodes were prepared from 80 wt.% LNMO powder, 10 wt.% carbon black, and 10 wt.% poly vinylidene fluoride (PVDF). N-methyl pyrrolidinone (NMP) was then added and ground evenly. Next, spread the mixture slurry evenly on the thin aluminum foil and vacuum dry at 120°C for 12 h. The 10 mm diameter electrode disc was then perforated from the coated foil. The LNMO/Li of 2,032 coins were assembled in argon filled ball cases with 2,400 Celgard dividers.

Electrochemical impedance spectroscopies (EIS) of LNMO/Li cells after 1 cycle, 2 cycles, and 3 cycles at 4.98 V were recorded by an electrochemical workstation (CHI660E, Chenhua, Shanghai), and the open-circuit voltages of the cells were set as the initial potential. The frequency range of LNMO/Li cells was 10,000~0.01 Hz. Cyclic voltammetry (CV) was detected by an electrochemical workstation with five cycles at a sweep rate of 0.1 mV s^−1^.

### Surface Detections of the LNMO Electrode

LNMO/Li cells after cycles were disassembled. First, the LNMO electrode was washed three times with high-purity DMC, and then transferred to a vacuum drying box at 45°C and placed in it for 4 h. The microstructure and morphology of LNMO electrode were recorded by scanning electron microscope (SEM). The surface morphology of LNMO electrode was detected though the transmission electron microscopy (TEM). X-ray photoelectron spectroscopy (XPS) was used to analyze the composition of chemical elements on the surface of LNMO electrode.

## Results and Discussion

### Cycling Performance Analyses

**Figure 1:**
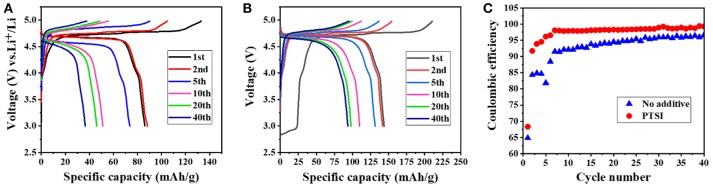
**(A,B)** Cycling performance of LNMO/Li cells at different cycles with no additive and with 0.5 wt.% PTSI additive in a voltage range of 3.0–4.98 V; **(C)** Coulombic efficiency of LNMO/Li cells with no additive and with 0.5 wt.% PTSI.

It can be clearly seen that the discharge specific capacity of LNMO/Li cell with 0.5 wt.% PTSI was higher than that without additives at the first cycle from in Figure 1. From in Figures 1A,B, the discharge specific capacity of LNMO/Li cell with additional PTSI reached 143.8 mAh/g, while the discharge specific capacity of the cell without additive is only 107.6 mAh/g. It can be concluded that PTSI additive could improve the initial discharge specific capacity of LNMO/Li cell. What's more, the specific capacity of LNMO/Li cell with 0.5 wt.% PTSI added after 40 cycles was much higher than that of LNMO/Li cell without additive. This also reflected that PTSI can indeed improve the specific capacity of LNMO/Li cell and provide a new scheme for improving the energy density of LIBs. In addition, the coulomb efficiency of the battery was constantly improved maintained a high level, as shown in Figure 1C. The coulomb efficiency of LNMO/Li cell without additives is 96% at the 40th cycle, while LNMO/Li cell with 0.5 wt.% PTSI added reached 99%. This indicated that the PISI additive can improve the coulomb efficiency during the charging/discharging cycle of LNMO/Li cell.

### Impedance Analysis

**Figure 2:**
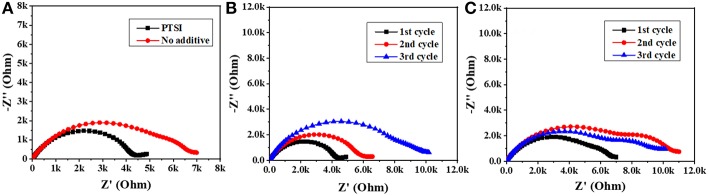
Impedance spectrum of LNMO/Li cells: **(A)** after 1 cycle; **(B)** the first three cycles with 0.5 wt.% PTSI; **(C)** the first three cycles with no additive.

In order to explore the interface impedance of SEI film between electrolyte and LNMO electrode, EIS of LNMO/Li cells with 0.5 wt.% PTSI and with no additive were recorded, as shown in Figure 2. In impedance spectroscopy, the semicircular high frequency region represents the migration of lithium ions through the interface at the surface of the LNMO electrode, and the center frequency range of the semicircle corresponds to the charge transfer process (Zhao et al., 2018). The results show that the interfacial impedance of LNMO electrode with 0.5 wt.% PTSI additive is significantly lower than that of LNMO electrode with no additive, suggesting that the surface of the LNMO electrode with 0.5 wt.% PTSI is improved. What is more, the two semicircles in the impedance spectra with 0.5 wt.% PTSI are significantly reduced compared with those with no additive. As the number of cycles increases, the impedance change of LNMO electrode with the addition of PTSI is much smaller than that of the LNMO electrode with no additive. In the lithiation/delithiation process, the surface layer impedance reduction and charge transfer will reduce ohmic polarization and activation polarization, which also confirms the above superior cyclic performance of the LNMO/Li cells with 0.5 wt.% PTSI.

### CV Measurements

**Figure 3:**
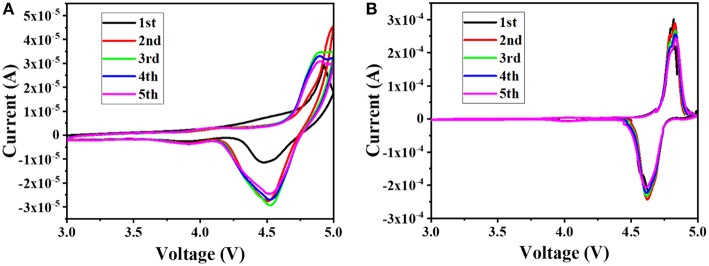
CVs of Li/LNMO cell with no additive **(A)** and with 0.5 wt.% PTSI **(B)**, at a sweep rate of 0.1 mV s^−1^.

In order to better understand the effect of the additive PTSI on the LNMO cell, the battery was subjected to the CV measurements at a sweep rate of 0.1 mV s^−1^ at 25°C, and the results are shown in Figure 3. It can be seen from the figure that there is a major redox peak at around 4.7 V, which corresponds to the redox process of Ni^2+^ and Ni^4+^(Talyosef et al., 2005). According to the comparison of Figures 3A,B, the peak current of the Li/LNMO cell to which 0.5 wt.% of PTSI was added is significantly larger than the peak current of the Li/LNMO cell without additives. In addition, the potential difference between the two peaks in the CV diagram of the LNMO cell to which PTSI was added is small, and as the number of cycles increases, the coincidence degree of the CV measurements pattern of the LNMO cells to which the PTSI was added is higher, indicating that the addition of PTSI makes the circulation of the LNMO cell more stable.

### SEM and TEM Analyses

**Figure 4:**
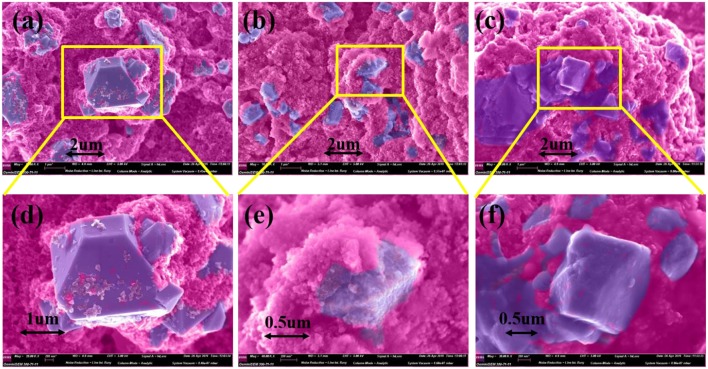
SEM images of LNMO electrodes with different electrolytes after 40 cycles: **(a,d)** fresh, **(b,e)** with no additive, and **(c,f)** with 0.5 wt.% PTSI.

To further study the effect of additive PTSI on the performance of LNMO electrode, SEM tests were carried out on the fresh electrode and the cycled LNMO electrode, as shown in Figure 4. The surface of the fresh electrode is very smooth and clean, without sediments, while the cycled LNMO electrodes show very different surface morphology. Compared to the fresh LNMO electrode, the surface of LNMO electrode with no additive is not smooth, which is coated with thick materials. Therefore, it increases the surface area, leading to an interface reaction that affects the transport of Li^+^ through the electrode. In contrast, the surface of the cycled LNMO electrode with 0.5 wt.% PTSI shows smooth and flat, forming thin materials on the surface. The relatively low viscosity of the additive PTSI improves permeability of electrolytes to LNMO electrode (Wang R. et al., 2015). Meanwhile, the reduction potential of PTSI was higher than carbonate solvents, which hinders the solvent decomposition in LiPF_6_-based electrolyte.

**Figure 5:**
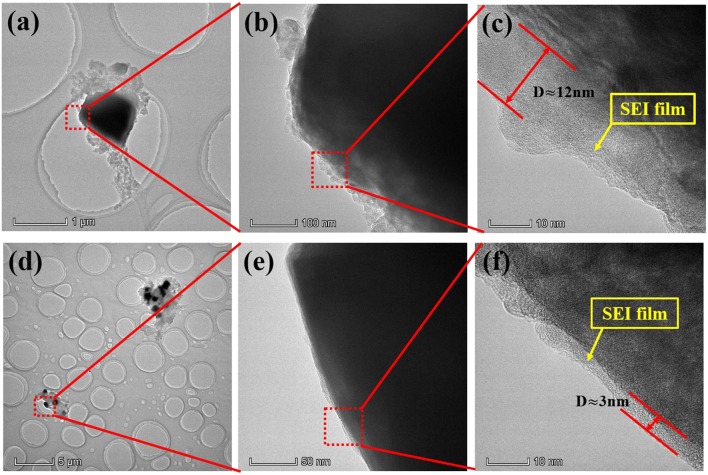
TEM images of LNMO electrodes after 40 cycles: **(a–c)** no additive and **(d–f)** with 0.5 wt.% PTSI.

In addition, in order to better describe the effect of additive PTSI on LNMO electrode, the corresponding TEM images of LNMO electrode after 40 cycles were obtained in Figure 5. It can be clearly seen that the LNMO electrode with 0.5 wt.% PTSI has a relatively clear layer boundary, which is the SEI film (~3 nm) generated on the surface of the electrode. The film is dense and uniform, which can effectively protect the LNMO electrode. However, a very uneven and thick SEI film (~12 nm) is generated on the LNMO electrode with no additive, which will slow down the transfer of Li^+^ between electrolyte and electrode. It markedly indicates that the decomposition of electrolyte, as well as the electrode erosion from the electrolyte with no additive, is more serious.

### XPS Analysis

**Figure 6:**
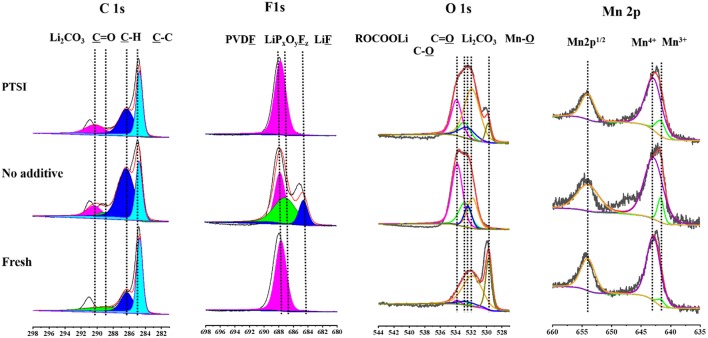
XPS survey spectra of LNMO electrode with 0.5 wt.% PTSI and with no additive after 40 cycles.

In order to verify the specific elements of surface layer about fresh electrode, non-additive electrode and the electrode with 0.5 wt.% PTSI after 40 cycles were detected by XPS in Figure 6. The C 1s spectra have four main peaks: C-C bond from the carbon black (284.1 eV), C-H bond roots in lithium alkyl carbonates (R-CH_2_OCO_2_-Li) and PVDF (286.0 eV), C=O bond belongs to lithium alkyl carbonates (R-CH_2_OCO_2_-Li) and polycarbonates (287.6 eV), and Li_2_CO_3_ (290.1 eV) (Funabiki et al., 1997; Levi et al., 2000; Dedryvere et al., 2010; An et al., 2016; Wang R. H. et al., 2015). It can be seen that the strength of Li_2_CO_3_ on the surface of LNMO electrode with 0.5 wt.% PTSI is significantly weaker than that on the surface of LNMO electrode with no additive, indicating the inhibitory effect of PTSI on electrolyte decomposition.

The O 1s spectrum displays five different peaks, including C-O peak (532.8 eV), Mn-O bond (529.7 eV), Li_2_CO_3_ (531.8 eV), C=O bond (532.4 eV), and C-O-C bond in lithium alkyl carbonates (R-CH_2_OCO_2_-Li) (533.8 eV) (Dedryvère et al., 2005; Bae et al., 2014; Wang et al., 2016). After PTSI was added, the C=O bond energy intensity increased, indicating that the polarization of EC and DEC solvents is effectively inhibited. In addition, Li_2_CO_3_ peak with no additive is stronger. That is, there are many inorganic decomposition products on the surface of the LNMO electrode with no additive.

In the F 1s spectrum, there is a significant difference between the two electrodes after cycling in different electrolytes. The peak strength of LiF (684.5 eV) and PVDF (687.7 eV) with 0.5 wt.% PTSI was significantly lower than that with no additive (Park et al., 2010; Zhou et al., 2011), indicating that there are few inorganic products on the electrode surface. When LiF content in the SEI film increasing, it will cause erosion to the electrode and inhibit the transport of Li ions. Hence, the impedance of LNMO electrode surface will increase accordingly. So, the SEI film generated by PTSI enhances the electrical charge transfer channel between LNMO electrode and electrolyte.

In the Mn 2p spectrum, there are three main characteristic peaks, which belong to Mn^3+^ (641.7 eV), Mn^4+^ (642.9 eV), and Mn 2p^1/2^ (653.6 eV) (Treuil et al., 1999). It is found that the Mn^3+^ peak strength of no additive electrolyte is lower than that of PTSI additive electrolyte, suggesting that HF causes the erosion of LNMO electrode surface.

In conclusion, SEM, TEM, and XPS indicate that the SEI film of LNMO electrode with 0.5 wt.% PTSI is thinner than that of LNMO electrode with no additive. The optimization of SEI film can greatly promote the transport of Li^+^ to a large extent and inhibit the oxidation decomposition of electrolyte, which can prevent the product from damaging the electrode.

### The Proposed Mechanism for LNMO/Electrolyte Interface Film

**Figure 7:**
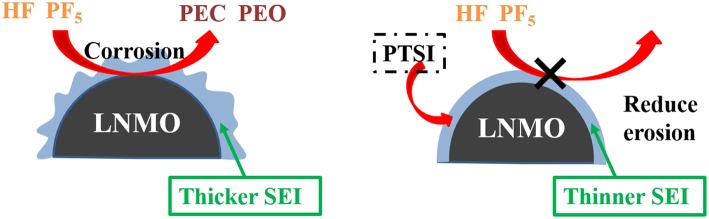
Schematic illustration of the effect of the electrolyte without and with PTSI additives on the LNMO cathode surface.

According to the above analysis, a schematic diagram of SEI film formation on the surface of LNMO electrode is obtained, as shown in Figure 7. Compared with the alkyl carbonic (Li_2_CO_3_ and ROCO_2_Li) generated by EC, the decomposition products (Li_2_SO_3_, Li_2_S, and ROSO_2_Li) formed by the additive PTSI are relatively more stable, which inhibits HF to corrode the surface of LNMO electrode and reduces interface impedance (Wang R. et al., 2017). Therefore, the diffusion of Li^+^ across the surface of LNMO was enhanced.

PF_5_ acted as a catalyst for the oxidation and corrosion of electrolyte, and could guide the reaction path of electrolyte to HF and H_2_O (Sloop et al., 2003). What's more, PF_5_ decomposed into EC and DEC. The open-loop reaction of EC was catalyzed by PF_5_, which leads to the polymerization of the reaction to produce polyethylene carbonate (PEC) and polyethylene oxide (PEO) similar products. As PF_5_ lack electrons, PTSI contains many electrons, including the S=O group, which caused the nitrogen nucleus to be delocalized and the weak base to be sited as inhibiting PF_5_ reactivity (Wu et al., 2012; Wang R. H. et al., 2015). The HF generated and LiF formed from LiPF_6_ will be inhibited. The SEI film formed on the surface of the LNMO electrode can reduce the interfacial resistance between LNMO and electrolyte.

What's more, PTSI played an important role in the development of SEI film, which successfully prevented HF from passing through the modified film to corrode the LNMO electrode. By reducing the reaction between PF_5_ and electrolyte, the content of LiF in the SEI film was reduced and the formation of HF was inhibited. The results show that PTSI can significantly inhibit the degree of oxidative decomposition of carbonate solvent during LNMO/Li cell cycle. It is concluded that PTSI is used as electrolyte additive for LNMO electrode at a high range voltage of 3.0–4.98 V.

## Conclusions

In this work, we report a electrolyte based on 1 M LiPF_6_ EC/EMC/DEC (1:1 by wt.%) with 0.5 wt.% PTSI for LNMO/Li. Electrochemical tests, EIS, CV, SEM, TEM, and XPS display that the decomposition of carbonate solvent has been inhibited and a dense SEI film on the electrode surface is formed. The electrolyte using PTSI as a non-aqueous electrolyte additive has good electrochemical stability at high voltages 4.98 V. The SEI film generated from PTSI is a stable protective layer, which inhibits HF erosion and reduces the interface resistance. As a result, LNMO/Li cells show excellent cycling performance.

## Data Availability

All datasets generated for this study are included in the manuscript/supplementary files.

## Author Contributions

ZX, RW, and ZQ designed and engineered the samples. ZX and RW performed the experiments. ZX, RW, YL, YS, SF, KX, HZ, and ZQ performed the data analysis. ZX, RW, and ZQ wrote the paper. All authors contributed to the theoretical analysis and the general discussion.

### Conflict of Interest Statement

The authors declare that the research was conducted in the absence of any commercial or financial relationships that could be construed as a potential conflict of interest.
